# Tolerance and rebound with zafirlukast in patients with persistent asthma

**DOI:** 10.1186/1477-5751-7-3

**Published:** 2008-05-19

**Authors:** David W Reid, Neil L Misso, Shashi Aggarwal, Philip J Thompson, David P Johns, E Haydn Walters

**Affiliations:** 1 Respiratory Research Group, Menzies Research Institute, University of Tasmania Hobart, Tasmania, Australia; 2 Lung Institute of Western Australia, Centre for Asthma, Allergy & Respiratory Research, The University of Western Australia, Perth, Australia

## Abstract

**Background:**

The potential for tolerance to develop to zafirlukast, a cysteinyl leukotriene (CysLT) receptor antagonist (LRA) in persistent asthma, has not been specifically examined.

**Objective:**

To look for any evidence of tolerance and potential for short-term clinical worsening on LRA withdrawal. Outcome measures included changes in; airway hyperresponsiveness to inhaled methacholine (PD_20_FEV_1_), daily symptoms and peak expiratory flows (PEF), sputum and blood cell profiles, sputum CysLT and prostaglandin (PG)E_2 _and exhaled nitric oxide (eNO) levels.

**Methods:**

A double blind, placebo-controlled study of zafirlukast, 20 mg twice daily over 12 weeks in 21 asthmatics taking β_2_-agonists only (Group I), and 24 subjects treated with ICS (Group II).

**Results:**

In Group I, zafirlukast significantly improved morning PEF and FEV_1_compared to placebo (p < 0.01), and reduced morning waking with asthma from baseline after two weeks (p < 0.05). Similarly in Group II, FEV_1 _improved compared to placebo (p < 0.05), and there were early within-treatment group improvements in morning PEF, β_2_-agonist use and asthma severity scores (p < 0.05). However, most improvements with zafirlukast in Group I and to a lesser extent in Group II deteriorated toward baseline values over 12 weeks. In both groups, one week following zafirlukast withdrawal there were significant deteriorations in morning and evening PEFs and FEV_1 _compared with placebo (p ≤ 0.05) and increased nocturnal awakenings in Group II (p < 0.05). There were no changes in PD_20_FEV_1_, sputum CysLT concentrations or exhaled nitric oxide (eNO) levels. However, blood neutrophils significantly increased in both groups following zafirlukast withdrawal compared to placebo (p = 0.007).

**Conclusion:**

Tolerance appears to develop to zafirlukast and there is rebound clinical deterioration on drug withdrawal, accompanied by a blood neutrophilia.

## Introduction

The cysteinyl leukotrienes (CysLTs), LTC_4_, LTD_4_, and LTE_4_, contribute to airway inflammation and bronchoconstriction in asthma [1-3]. Cysteinyl leukotriene receptor antagonists (LRAs) and synthesis inhibitors are widely used as anti-asthma therapies and they have been convincingly shown in research studies to improve lung function and clinical status as well as reduce exacerbation rate and airway inflammation. However, in clinical practice, therapeutic response is difficult to predict and quite variable. Head to head studies have confirmed that inhaled corticosteroids (ICS) and ICS/long-acting β_2_-agonist combinations are superior to the LRAs in achieving clinical control and the place of LRAs in asthma management guidelines remains uncertain [4-7]. Studies of LRAs have confirmed their safety and this is one of the attractions compared to ICS therapy, but no studies have specifically looked for evidence of tolerance or rebound deterioration on drug withdrawal.

Zafirlukast (Accolate^®^, Astra Zeneca) is a highly selective LTD_4 _antagonist [8]. The primary objective of this study was to determine whether the clinical benefits of zafirlukast 20 mg twice daily (b.d) would be sustained over 12 weeks treatment and whether there was any potential for short-term deterioration in asthma control following drug withdrawal. We were secondarily interested in whether clinical benefits were related to any potential anti-inflammatory effects of zafirlukast and whether these would similarly deteriorate on drug cessation. Treatment was assessed in two distinct groups of subjects with persistent asthma: in symptomatic subjects maintained on β_2_-agonists alone and in subjects with persistent asthma symptoms despite moderate doses of ICS. Both of these asthmatic groups are ones in which clinicians may consider the use of a LRA.

## Methods

### Subjects (Table 1)

**Table 1:** Patient demographics at baseline

	β_2_-agonists + Placebo (N = 7)	β_2_-agonists + Zafirlukast (N = 14)	ICS-treated + Placebo (N = 8)	ICS-treated + Zafirlukast (N = 16)
Sex, male/female	3/4	8/6	2/6	9/7
Age, years	29 (21–55)	42 (21–69)	45 (30–65)	37 (19–65)
Ex-smoker	2	4	5	6
FEV_1_, L	2.7 (2.3–3.7)	2.1 (1.4–4.2)	2.8 (2.0–3.8)	2.7 (1.4–4.1)
Baseline FEV_1_, % predicted	80 (65–102)	85 (69–107)	76 (60–95)	77 (56–98)
Inhaled corticosteroid, μg/day	NA	NA	1600 (1000–2400)	1600 (1000–2400)
PD_20 _methacholine, μg *	0.008 (0.001–0.04)	0.04 (0.005–1.3)	0.03 (0.004–0.2)	0.02 (0.001–0.6)

Data are given as median and (range) except * geometric mean and (range). NA not applicable

Non-smoking adult subjects with a history of at least one year of persistent asthma symptoms treated with either β_2_-agonists alone (Group I) or β_2_-agonists plus moderate/high dose of ICS (≥ 800 μg Budesonide or equivalent daily), for a minimum period of four weeks (Group II) were eligible for participation. Exclusion criteria included: history of an asthma exacerbation, upper respiratory tract infection or alteration in asthma medication within six weeks, or use of oral corticosteroids within three months of screening. Patients were also excluded if they had received a long-acting β_2_-agonist (LABA), anticholinergic, cromone or theophylline during the six weeks prior to the screening visit. Volunteers were recruited through advertisement. The study was approved by the Alfred Hospital's Research Ethics Committee and written informed consent was obtained from each person.

### Study design (Figure 1)

**Figure 1:**
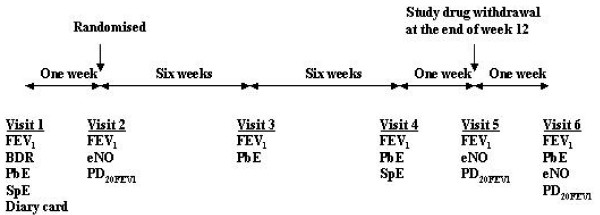
Study Design. BDR bronchodilator reversibility, PbE peripheral blood eosinophils, SpE sputum eosinophils, eNO exhaled nitric oxide levels.

This was a 13 week, single centre, randomised, double blind, placebo-controlled study. A pre-study visit to confirm selection criteria was followed by a second visit for randomisation after a one-week screening period. Figure 1 gives details of investigations and procedures performed at each study visit. To be eligible, subjects had to demonstrate significant bronchodilator reversibility (BDR) i.e. ≥ 15% increase in FEV_1 _after 400 μg of salbutamol or significant diurnal PEF variability (≥ 15%) during the run-in period. All subjects had to have a baseline FEV_1 _of ≥ 60% predicted after withholding inhaled β_2_-agonists for six hours. Before randomisation, subjects needed a minimum cumulative symptom score (asthma severity score) of ≥ 10 (maximum 21), over the last seven days of the screening period using a daily three point scale; 0 = no symptoms, 1 = mild symptoms not interfering with activities, 2 = moderate symptoms interfering with some activities, 3 = severe symptoms interfering with most activities.

Eligible subjects were randomised to either zafirlukast 20 mg b.d or placebo b.d on a two to one basis using a computer-generated random number scheme by the hospital research pharmacist, who then dispensed zafirlukast and placebo as identical tablets in identical blister packs.

Subjects withheld inhaled β_2_-agonists for six hours and study medication on the morning of each visit except for visit five (see below). Spirometry was performed at every visit using a calibrated electronic spirometer (MedGraphics, Minneapolis, Minenesota US) and the best of three technically acceptable FEV_1 _measurements was recorded.

### Clinical outcome measures

Daily asthma symptom scores, relief medication use and nocturnal awakenings were recorded on a diary card. Morning and evening PEF (best of three) was also recorded each day before use of inhaled β_2_-agonist or administration of study medication. For analysis, diary card entries were assessed at three weeks, six weeks, at the end of active treatment (week 13) and following study drug withdrawal (week 14).

### Airway hyperresponsiveness and indices of inflammation

#### Methacholine challenge

AHR to inhaled methacholine challenge was performed according to a standardised protocol. [9] Results were expressed as PD_20FEV1_, the cumulative dose of methacholine estimated to provoke a 20% decrement in FEV_1 _determined by linear interpolation between the last two points on the dose-response curve. At visit 5, the bronchoprotective effect of zafirlukast *vs *placebo was determined by performing methacholine challenge two hours after observed administration of the morning dose of study drug.

#### Sputum induction and processing

Subjects were pre-medicated with inhaled salbutamol 400 μg and after 15 minutes they inhaled hypertonic saline (4.5%, DeVilbiss Ultrasonic Nebuliser, Jackson, Tennessee) for five minutes before being asked to expectorate sputum. Before coughing, saliva was discarded to minimise buccal contamination. This procedure was repeated to a maximum of six nebulisations. FEV_1 _was measured if the patient felt uncomfortable and sputum induction was terminated when the subject had expectorated ≥ 2.5 mLs of sputum with visible airway "plugs". Following sputum induction, FEV_1 _was measured and salbutamol administered if FEV_1 _was ≤ 80% of the pre-induction value.

Whole sputum sample processing and cell counting was performed according to the methods of Fahy [10]. Briefly, a volume of dithiothreitol 0.1% (Sputalysin; Calbiochem Ltd. CA, USA) equivalent to four times the weight of sputum was added. The sample was placed in a water bath at 38°C for 30 minutes and mixed at intervals to ensure adequate homogenisation. The sample was then centrifuged (Shandon II cytocentrifuge) at 1500 rpm for 10 minutes and cell-free supernatant decanted and stored at -80°C (see later). The cell pellet was resuspended with phosphate buffered saline to the original sputum volume. A total cell count was performed in a Neubauer hemocytometer and the resuspended sample spun in a cytocentrifuge (Shandon cytospin III, Runcorn, UK; 82 g) for 10 minutes. Cytospots were stained with Diff-Quik and two slides per sputum sample were analysed by an observer blinded to subject. At least 200 non-squamous cells were counted on each slide and the results averaged. A sputum sample was considered adequate if the percentage of squamous cell contamination was less than 80% [11].

#### Total cysteinyl leukotriene and prostaglandin E_2 _assays

CysLT and PGE_2 _were extracted from induced sputum supernatants by immunoaffinity purification using affinity sorbents (mouse monoclonal cysLT or PGE_2 _antibody covalently bound to Sepharose 4B, Cayman Chemical, Ann Arbor, MI, USA). After thawing, 0.2 ml of sputum supernatant was incubated with 20 μl of cysLT affinity sorbent or 50 μl of PGE_2 _affinity sorbent with gentle mixing for 1 h at room temperature. After centrifugation (10,000 rpm, 4 min), the supernatant was discarded and the sorbent pellet was washed with 1 ml of PBS. CysLT or PGE_2 _were then eluted from the sorbents with 1 ml of methanol or 95% ethanol, respectively. The methanol or ethanol extracts were evaporated to dryness under vacuum and then resuspended in enzyme immunoassay buffer. Total CysLT (LTC_4_/LTD_4_/LTE_4_) and PGE_2 _concentrations were determined with specific enzyme immunoassay kits according to the manufacturer's instructions (Cayman Chemical). Using this methodology, processing with DTT has been shown to have no effect on detectable levels of sputum CysLT or PGE_2 _[12]. Recoveries of cysLT and PGE_2 _through the extraction and enzyme immunoassay were assessed by spiking sputum supernatants with known amounts of LTD4 and PGE_2_, with the unspiked samples being assessed in parallel for endogenous concentrations of cysLT and PGE_2_. The mean recovery of LTD4 was 65.5% ± 10.9% (SEM, n = 8) and the mean recovery of PGE_2 _was 115.8 ± 8.8% (SEM, n = 8).

#### Exhaled breath nitric oxide determination

NO measurements were obtained using the method described by Silkoff with patients inhaling NO-free gas (Medical Air, Air Liquide Australia, Melbourne) and exhaling against a fixed resistance to ensure closure of the soft palate [13].

Exhaled NO (eNO) was measured using a fast response, high sensitivity chemiluminescence analyser (Sievers NOA 270 B, Boulder, Colorado, USA) with a lower detection limit for NO of 0.3 parts per billion (ppb).

The mean concentration of the plateau phase of the single breath test was recorded from 3 technically acceptable measurements.

### Statistical Analysis

Independent professional statistical advice was obtained for the analysis. Analyses were performed according to the distribution of the data with or without log transformation. Clinical data are expressed as least square means with standard errors of the means (SEM). Sputum and blood results are expressed as median and range. Changes in diary card and lung function parameters with treatment were compared using a repeat measures analysis of co-variance (ANCOVA) with the mean of the variables for the last seven days of the diary card during run-in as a covariate. Changes in diary card entries were assessed based on the mean recordings for the last 14 days of the study treatment periods between baseline and five weeks and between six weeks and 10 weeks (inclusive). The mean diary card recordings for the week prior (week 11) to study drug withdrawal at week 12 were then compared to baseline and the mean recordings for the one-week post-withdrawal. Within-treatment group changes from baseline for normally distributed data were assessed using paired t-tests. If there appeared to be a deviation from normality, statistical analysis was repeated using Wilcoxon's sign rank test to confirm the ANCOVA. Sputum and blood results were analysed according to the non-normal distribution of the data: Mann-Whitney U test was used to test differences between treatments and Wilcoxon was used to determine within-treatment group effects. Analyses were based on an intention to treat (ITT) principle wherever data were available, in order to allow several minor protocol violators to be included in-spite of the danger of positive signals being diluted. AHR data are presented as geometric means and ranges for PD_20FEV1_. Changes from baseline for PD_20FEV1 _values after acute dosing and following washout are expressed as a doubling concentration dose of methacholine using the following formula:

[Log_10 _PD_20FEV1 _(treatment) - Log_10 _PD_20FEV1_(baseline)]/log_10_2

The study was designed to detect a doubling dose difference of 1.0 in PD_20FEV1 _between weeks 0 and 12 between the treatment arms in each asthmatic subpopulation studied (12 patients on zafirlukast and six on placebo) with 80% power. Correlations between categorical variables were analysed using Spearman's rank test. Statistical analyses were undertaken in SPSS with a two-tailed p ≤ 0.05 being considered statistically significant.

## Results

### Adverse events and withdrawals

Twenty-one subjects using β_2_-agonists alone (median age 41 years, range 21–69 years, 10 female; Group I), and 24 asthmatic subjects maintained on ICS (median age 42 years, range 19–65 years, 14 female; Group II), met the entry criteria for the study.

In Group I, of 14 subjects randomised to zafirlukast, one withdrew consent shortly after randomisation for personal logistic reasons and another subject withdrew for similar reasons after four weeks treatment. One subject developed an upper respiratory tract infection and asthma worsening following cessation of zafirluklast and was unable to undergo repeat determination of eNO levels at visit six. One additional subject did not complete their diary card following zafirlukast withdrawal, because this occurred over the Christmas period. Two subjects completed the study but did not undergo methacholine challenge at visit five: one subject was unable to withhold rescue medication for six hours prior to testing, and FEV_1_deteriorated to < 60% predicted pre-test in another subject, thus precluding methacholine challenge. Of the seven subjects in Group I randomised to placebo, one withdrew consent shortly after randomisation and one subject completed the study but did not undergo methacholine challenge at visit five because of worsening lung function. Twelve subjects in Group I randomised to zafirlukast and five subjects in the placebo arm therefore completed the entire active treatment phase.

In Group II, three subjects of the 16 initially randomised to zafirlukast did not complete the treatment phase of the study; one subject because of worsening rheumatoid arthritis, one was found to have been inappropriately randomised because of neutropenia at screening and one subject developed angina necessitating cardiology referral. Two of these subjects completed the first four weeks of the study and their data were therefore entered into the prospective analyses. Following cessation of zafirlukast in Group II, two subjects developed clinical asthma worsening and one of these suffered a frank asthma exacerbation requiring oral corticosteroids. Outcome analysis in this sub-population was therefore based on the 15 subjects randomised to zafirlukast and eight randomised to placebo.

Adherence was assessed at each study visit by tablet counting and was found to be greater than 90% in all volunteers for the duration of the study.

### Clinical outcomes

In both groups overall, there was a pattern of improvement over the first two weeks with zafirlukast, but these changes then deteriorated back to baseline, or below baseline, by 12 weeks, with further deterioration or even frank exacerbation in one individual, in the withdrawal period. These changes were most marked in Group I, whereas in Group II there was some confounding by more general trends toward improvement in both active and placebo arms, probably related to "trial-induced" improvement in adherence to ICS therapy.

In Group I, initial improvement compared to placebo was most marked for laboratory-measured FEV_1 _and home recorded morning PEF (p < 0.01), with borderline levels of significance for early improvements in total mornings per week awakening with asthma (p = 0.03), total awakenings with asthma (p = 0.09) and β_2_-agonist use (p = 0.06) for within-treatment group comparisons. Following withdrawal of zafirlukast there were significant deteriorations in FEV_1 _and evening PEF compared to placebo (p = 0.05, p = 0.03, respectively). Additionally, within-treatment group comparisons also revealed a significant deterioration in morning PEF (p < 0.001) for zafirlukast.

In Group II, only FEV_1 _improved significantly (p = 0.04) after 2 weeks treatment with zafirlukast compared to placebo. Within-treatment group comparisons revealed significant improvements in morning PEF (p = 0.01), β_2_-agonist use (p = 0.02) and asthma severity scores (p = 0.02), and a trend toward improvements in evening PEF (p = 0.08). There were subsequent deteriorations in the group as a whole in morning and evening PEFs and mornings waking with asthma after six weeks of treatment compared to baseline. Following withdrawal of zafirlukast, FEV_1 _deteriorated significantly compared to placebo, (p = 0.04), and there were within-treatment group rebound deteriorations in evening PEF (p < 0.005) and nocturnal awakenings (p = 0.04).

**Figure 2:**
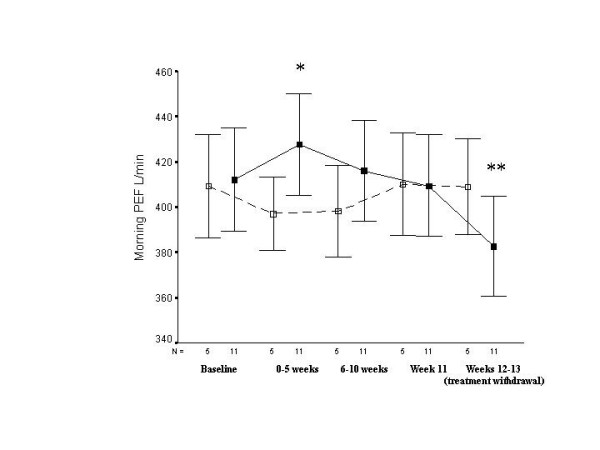
Effects of zafirlukast on morning PEF in asthmatic subjects maintained on β_2_-agonists alone. Solid line represents zafirlukast and dashed line, placebo. Only data from subjects completing the entire study are illustrated. *p = 0.01 compared to placebo, **p < 0.001 compared to before withdrawal.

Overall, with both groups combined, these changes in morning and evening PEF and FEV_1 _reached statistical significance compared to placebo (p < 0.05). Thus, of particular note was the quite definite deterioration in both asthmatic groups on cessation of zafirlukast in a range of indices. These general trends are illustrated by the changes that occurred in morning PEF in Group I (figure 2), but these are reasonably typical of physiological and clinical changes across the board (tables 2 &3). All together, there were five clinical exacerbations on stopping active medication, and none on stopping placebo. One of the subjects in Group II required a short-course of rescue oral CS following zafirlukast withdrawal.

**Table 2:** Effects of zafirlukast in asthmatic subjects maintained on β_2_-agonists alone

	β_2_-agonists alone + Placebo Change from baseline					β_2_-agonists alone + Zafirlukast Change from baseline				
	Baseline (N = 7)	Weeks 0–5 (N = 6)	Weeks 6–10 (N = 5)	Week 11–12 (N = 5)	*W/D (N = 5)	Baseline (N = 14)	Weeks 0–5 (N = 13)	Weeks 6–10 (N = 12)	Week 11–12 (N = 12)	*W/D (N = 11)

Daily PEFR a.m., L/min	375 (± 36.7)	-17.3 (± 8.6)	-12.2 (± 10.3)	+0.8 (± 7.5)	-1.2 (± 4.3)	408 (± 30.9)	+14.9 (± 7.9)	+1.6 (± 8.5)	-3.2 (± 11.5)	-30.7 (± 10.9)
Daily PEFR p.m., L/min	374 (± 35.0)	+4.9 (± 6.6)	+14.2 (± 10.7)	+21.2 (± 11.7)	-9.4 (± 8.9)	430 (± 27.4)	+4.2 (± 4.4)	-0.08 (± 5.7)	-5.8 (± 7.5)	-12.7 (± 8.2)
FEV_1_, mL	2.82 (± 0.33)	-170 (± 80)	+20.0 (± 10)	-258 (± 213)	+7.2 (± 128)	2.90 (± 0.16)	+110 (± 60)	-12.5 (± 6.9)	+14.2 (± 72)	-214 (± 69.8)
β_2_-agonist use per day	4.6 (± 0.9)	-0.7 (± 0.3)	-1.3 (± 0.4)	-0.6 (± 0.5)	+0.2 (± 0.5)	3.7 (± 0.5)	-0.7 (± 0.4)	-0.4 (± 0.5)	+0.2 (± 0.5)	+0.6 (± 0.3)
Severity score	1.9 (± 0.05)	-0.2 (± 0.1)	-0.3 (± 0.1)	-0.2 (± 0.2)	-0.04 (± 0.2)	1.8 (± 0.08)	-0.2 (± 0.1)	-0.2 (± 0.1)	-0.2 (± 0.2)	+0.3 (± 0.2)
**Total mornings	4.1 (± 1.0)	-0.07 (± 0.5)	-0.2 (± 0.3)	-0.8 (± 0.4)	-0.2 (± 0.5)	3.5 (± 0.7)	-1.1 (± 0.5)	-0.8 (± 0.6)	-0.3 (± 0.7)	+0.1 (± 0.5)
**Total awakenings	3.7 (± 1.3)	-1.1 (± 0.7)	-0.3 (± 1.7)	+0.7 (± 2.4)	-0.2 (± 0.7)	1.4 (± 0.5)	-0.8 (± 0.4)	-0.9 (± 0.5)	+0.2 (± 0.7)	+0.9 (± 0.8)

**Table 3:** Effects of zafirlukast in subjects maintained on ics

	ICS-treated + Placebo Change from baseline					ICS-treated + Zafirlukast Change from baseline				
	Baseline (N = 8)	Weeks 0–5 (N = 8)	Weeks 6–10 (N = 8)	Week 11–12 (N = 8)	*W/D (N = 8)	Baseline (N = 15)	Weeks 0–5 (N = 15)	Weeks 6–10 (N = 13)	Week 11–12 (N = 13)	*W/D (N = 11)

PEFR a.m., L/min	353 (± 32.3)	+13.9 (± 6.1)	+21.1 (± 9.6)	+17.5 (± 12.2)	+10.4 (± 6.6)	389 (± 21.2)	+15.7 (± 5.0)	+16.7 (± 6.1)	+2.7 (± 15.2)	-7.4 (± 6.3)
PEFR p.m., L/min	373 (± 32.8)	+9.4 (± 10.0)	+6.4 (± 15.0)	+17.7 (± 12.1)	+5.3 (± 6.2)	404 (± 20.0)	+10.5 (± 4.9)	+10.9 (± 5.8)	-0.6 (± 15.0)	-23.1 (± 6.6)
FEV_1_, mL	2,567 (± 225)	-134 (± 67.7)	-130 (± 47.2)	-23.8 (± 93.6)	+78.8 (± 59.6)	2,540 (± 179)	+105 (± 72.5)	-39.2 (± 74.7)	+144 (± 106)	-94.5 (± 48.0)
β_2_-agonist use per day	3.9 (± 0.6)	-0.8 (± 0.47)	-1.2 (± 0.68)	-0.3 (± 1.0)	-0.4 (± 0.4)	3.7 (± 0.5)	-0.8 (± 0.25)	-0.9 (± 0.32)	-1.1 (± 0.4)	+0.6 (± 0.47)
Severity score	2.0 (± 0.02)	-0.3 (± 0.1)	-0.3 (± 0.1)	-0.03 (± 0.04)	-0.1 (± 0.1)	1.8 (± 0.7)	-0.3 (± 0.1)	-0.3 (± 0.1)	-0.4 (± 0.2)	+0.1 (± 0.2)
**Total mornings	5.7 (± 0.8)	-1.8 (± 0.8)	-3.2 (± 0.9)	-2.3 (± 1.4)	-0.8 (± 2.2)	3.2 (± 0.6)	-0.7 (± 0.4)	-1.1 (± 0.7)	-0.6 (± 0.5)	+0.4 (± 0.3)
**Total awakenings	1.5 (± 0.9)	-0.8 (± 0.7)	-0.8 (± 0.5)	-1.1 (± 0.7)	+0.4 (± 1.1)	1.8 (± 0.7)	-0.6 (± 0.5)	-0.9 (± 0.6)	-1.6 (± 0.7)	+1.4 (± 1.0)

In both groups, there was no relationship whatsoever between deterioration on zafirlukast withdrawal and the initial improvements observed when treatment was instituted i.e. those who deteriorated the most were not those who had derived the greatest initial benefit with zafirlukast.

### Methacholine challenge

AHR at baseline was similar in both asthmatic groups (Table 1). We were unable to assess treatment differences in Group I because of the small numbers who underwent repeated testing at the end of active treatment and following withdrawal. However, comparison of Group I baseline results to those after acute active dosing at visit 5 revealed a trend for geometric mean PD_20FEV1_to rise from 0.038 μg to 0.057 μg, representing a 0.6 doubling-dose (DD) improvement (p = 0.07).

In Group II, the effect of zafirlukast was not significantly different to placebo, with PD_20 _methacholine improving in both treatment arms. Within-treatment group analysis demonstrated a small but significant rise in geometric mean PD_20FEV1 _from 0.025 μg at baseline to 0.042 μg (DD of 1.3; p < 0.05), after acute active dosing at visit 5, but this was not different to placebo given at the same time.

There was no significant deterioration in PD_20FEV1 _in either asthmatic group following zafirlukast withdrawal. Indeed, in Group II, comparing end of washout with pre-study test results revealed a persisting improvement in PD_20FEV1 _of 1.2 DD, which was significant (p < 0.05), but changes with placebo were similar though smaller. These changes probably reflect improved adherence with ICS therapy.

### Induced sputum and peripheral blood cell differentials (Tables 4 &5)

**Table 4:** Sputum and peripheral blood cellular profiles at baseline

	**β_2_-agonist alone**	**ICS-treated**	**Significance**
*TCC × 10^3^**/ml**	41.5 (11.1–235.3)	30.0 (0.5–293.8)	NS
**Sputum eosinophils, %**	4.6 (0.8–20.4)	6.0 (0.2–39.2)	NS
**Cys-LT pg/ml**	266.4 (1.0–1343.6)	297.6 (60.3–763.1)	NS
**PGE**_2_**pg/ml**	966 (371.6–1950.5)	1358,5 (149.1–6155.3)	NS
**PB eosinophils, × 10**^9^	4.3 (1.1–12.7)	3.3 (1.2–9.8)	NS
**PB neutrophils, × 10**^9^	3.2 (2.0–6.2)	3.65 (1.1–6.2)	NS
**eNO (ppb)**	31 (9–77)	15 (8–32)	p < 0.001

**Table 5:** Effects of zafirlukast on sputum inflammatory indices in paired samples

	**Zafirlukast (n = 9)**		**Placebo (n = 11)**	
	**Baseline**	**12 weeks**	**Baseline**	**12 weeks**
*TCC × 10^3^**/ml**	38.8 (0.6–293.8)	22.8 (0.3–93.8)	41.0 (0.5–176)	41.5 (3.8–109.3)
**Sputum eosinophils, %**	5.8 (0.8–39.2)	4.0 (0.4–54.6)	5.3 (0.2–14.7)	1.7 (0–39.5)
**CysLT pg/ml**	331 (182–648)	567 (151–1029)	192 (15–763)	412 (49–1332)
**PGE_2 _pg/ml**	942 (572–1951)	1276 (836–2513)	1245 (479–6155)	959.2 (329–3756)

*Data are expressed as median and (range).

Sputum eosinophils and PbE were closely related to each other in both asthmatic groups at baseline (r_(s) _= 0.8 & r_(s) _= 0.8, p < 0.001 respectively). Satisfactory baseline sputum samples were obtained from 31 subjects (Group I; n = 15, Group II, n = 16), and 24 subjects provided adequate paired samples (Group I; n = 13, Group II, n = 11). For sputum analysis we have therefore combined the groups (zafirlukast; n = 13). Paired blood samples were available in 37 subjects overall (zafirlukast; n = 23). There were no significant changes in either SpE or PbE numbers with 12 weeks treatment with zafirlukast, and there was also no increase in PbE on withdrawal of zafirlukast.

Zafirlukast treatment had no effect on sputum or peripheral blood neutrophils, but following withdrawal there was a significant rise in median number of circulating neutrophils in both groups overall compared to placebo (3.2 × 10^9^/L, range 1.6–4.2 × 10^9^/L to 3.5 × 10^9^/L, range 1.7–5.1 × 10^9^/L *versus *4.1 × 10^9^/L, range 2.8–5.4 × 10^9^/L to 3.4 × 10^9^/L, range 2.2–5.5 × 10^9^/L respectively, p = 0.007). This was most marked in the β_2_-agonist only group when post-withdrawal neutrophils rose uniformly compared to end of active treatment numbers; 3.0 × 10^9^/L (range 1.9–4.2 × 10^9^/L) to 3.7 × 10^9^/L (range 2.2–5.0 × 10^9^/L, p = 0.005). There was no statistical relationship between the observed changes in blood neutrophils and clinical deteriorations on zafirlukast withdrawal.

### Sputum CysLT and PGE_2 _levels (Table 5)

Sufficient paired sputum supernatants for analysis were available in 20 subjects. There was a trend for increased CysLT levels in Group I at baseline (p = 0.08), but no difference in PGE_2 _concentrations. Paired sputum supernatants were available for analysis in nine subjects who received zafirlukast (Group II; n = 3) and 11 subjects who received placebo (Group II; n = 7). Overall, with both groups combined there was no suggestion of a treatment effect on CysLT levels. Similarly, there was no suggestion that zafirlukast affected sputum PGE_2 _levels or the CysLT/PGE_2 _ratio.

CysLT and PGE_2 _levels at baseline and at the end of treatment were not related to any of the observed clinical outcomes in those subjects who received zafirlukast.

### Exhaled nitric oxide levels

As expected, eNO levels were significantly higher in Group I asthmatics (median 31 ppb, range 9–77 ppb) compared to Group II (15 ppb, 8–32 ppb) at baseline (p < 0.0001).

There were no changes in eNO levels in either asthmatic group following treatment with zafirlukast and no rise in eNO following treatment withdrawal.

## Discussion

Our findings demonstrate that early clinical improvements in asthmatic subjects treated with zafirlukast gradually wane to baseline values over a 12 week treatment period. Asthma control then significantly deteriorated overall when zafirlukast was withdrawn, i.e. to worse than baseline in many patients, especially in those not treated with an ICS. The changes with treatment and on withdrawal were less obvious in asthmatic subjects maintained on moderate doses of ICS (Group II), but the parallel improvement in the placebo group in this sub-population suggest our findings were probably confounded by improved adherence to ICS therapy as tends to occur during research studies. There was no evidence of an anti-inflammatory effect for zafirlukast, but drug withdrawal was accompanied by a significant increase in circulating neutrophils in both asthmatic groups. The changes with zafirlukast in both asthmatic populations were remarkably consistent and strongly suggest the development of tolerance and rebound deterioration on treatment withdrawal.

The leukotriene receptor antagonists (LRAs) have been attributed with potential disease modifying effects, although most of the evidence for an anti-inflammatory effect comes from studies of montelukast. [14-16] No previous direct evidence of tolerance to the LRAs has been presented in persistent asthma, although studies of exercise-induced asthma do suggest that tolerance can develop, but what may be most important is the dose and type of LRA used. [17] Ours is the first clinical study to prospectively demonstrate this possibility in persistent asthma and the challenge is to explain these findings in the light of large studies that have failed to find such an effect. [17,18]

The majority of studies of zafirlukast have been undertaken over short periods of six weeks or less, which may not have allowed sufficient time for tolerance or tachyphylaxis to develop or to be recognised. In our study, definite loss of clinical benefit generally occurred from the sixth week of treatment onward in both asthmatic groups, suggesting shorter treatment courses may not allow enough time for tolerance to occur. Although a handful of studies have demonstrated zafirlukast 20 mg bid over 12 weeks and longer to be significantly superior to placebo, the majority of clinical benefit has usually occurred within four weeks with little change thereafter. [19-21] Only larger than conventional doses of zafirlukast have shown improvements consistent with an anti-inflammatory effect, but whether tolerance develops to such aggressive dosing regimes remains unknown. [5]

The significant physiological deteriorations to below baseline values, especially in Group I, on zafirlukast withdrawal are inconsistent with simple removal of a bronchodilator effect, especially as FEV_1 _and PEF in most subjects had already deteriorated back to baseline values by the end of active treatment. There was a clear "over-shoot" to worse than study entry values in essentially all lung function and clinical parameters. There was also no relationship between initial improvements and subsequent deteriorations on zafirlukast withdrawal, so they do not seem predictable. A drop-off in adherence with zafirlukast over the 12 weeks of the study is unlikely to be an explanation for the gradual loss of benefit, as we assessed this very carefully at each study visit and the rebound deterioration observed only in subjects who received zafirlukast, which would be very much against poor adherence with active treatment.

We found no evidence of an anti-inflammatory effect for zafirlukast, despite our assessment of sputum and peripheral blood eosinophils as well as AHR and eNO levels. This would be against any worsening of inflammation related to some "masking" effect to explain the loss of clinical benefit over time. Following zafirlukast withdrawal there was a significant increase in peripheral blood neutrophils, especially in the β_2_-agonist group. Neutrophils express cysLT1 receptors and montelukast has been shown to reduce sputum neutrophils in stable COPD and there are also suggestions that neutrophil function may be modulated by LRAs. [22-27]

Acute dosing with zafirlukast appeared to confer some protection against methacholine-induced bronchoconstriction in both asthmatic groups, but this was not significant compared to the effects of placebo and our study was handicapped by the number of subjects, especially in the zafirlukast arm, who did not undergo repeat challenge testing at the end of active treatment or following withdrawal because of clinical deterioration. However, despite the small numbers available for analysis, PD_20FEV1 _did not deteriorate in either group following withdrawal of zafirlukast, suggesting that any acute protective effects were small and that LTD_4 _hypersensitivity was not manifest as increased AHR to methacholine.

One potential explanation for the clinical deteriorations over time in those subjects receiving zafirlukast would be up-regulation of LTD_4 _receptor expression in the airways, including on smooth muscle cells, induced by chronic receptor occupation by the LRA. Our data suggest that this may occur irrespective of clinical benefit. The lesser "tachyphylaxis" and rebound in Group II suggests ICS may protect against this, but the confounding of better adherence with disease-modifying ICS makes differences difficult to interpret. If LTD_4 _receptor expression is indeed up-regulated, then concomitant failure to reduce CysLT production by airway eosinophils could result in excessive receptor occupancy and activation on treatment withdrawal. The existence of this sort of dynamic receptor regulation is well described with histamine (H_2_)-receptor antagonists and explains the development of tolerance in peptic ulcer disease and rebound acid hypersecretion on drug withdrawal. H_2_-receptor antagonists demonstrate "inverse agonist" activity which leads to increased H_2_-receptor cell-surface expression. A recent short-term cell-culture model has demonstrated that zafirlukast and montelukast both function as reverse agonists causing cells to increase surface expression of CysLT_1 _receptors. This effect is likely to be much greater with longer-term exposure to LRAs. The importance of these observations is that they support the existence of dynamic cell-surface CysLT_1 _receptor expression and lend biological plausibility to our explanation for the loss of asthma control over time and rebound on LRA withdrawal. [28,29] Tachyphylaxis to β_2_-agonist therapy is another example of the potential for dynamic receptor expression, although in this context the effect is in the opposite direction with down-regulation of cell surface receptors following long-term exposure to agonist therapy. [30] Interestingly, ICS are known to modulate the development of tachyphylaxis to β_2_-agonists and although speculative, perhaps ICS also affect CysLT_1 _receptor expression to explain the lesser evidence of tolerance to zafirlukast in the ICS treated group observed in our study, but this requires further investigation. [31]

The question still remains as to whether our findings indicate a "class effect" or whether this is more likely with zafirlukast. The *in vitro *demonstration of up-regulated CysLT_1 _receptor expression with zafirlukast and montelukast would suggest a class effect, but several long-term clinical studies of montelukast have not suggested tolerance. [32] However, there are a number of reasons why tolerance could be masked: 1) it was not specifically looked for; 2) the population on average may not decline sufficiently for the effect to become clinically obvious, especially if improvements in a responsive sub-population counter-balance deteriorations in the remainder. The majority of absolute changes (deteriorations) in clinical status and lung function seen in our subjects treated with zafirlukast were quite small, albeit real, and would support this explanation. Additionally, drop-outs due to deterioration would reinforce this false impression of well-being in the "survivor population"; 3) ICS might modify the effect or patients may increase the ICS dose to counter any negative effects of tachyphylaxis that appear; 4) higher doses of LRAs may overcome the effect; and finally, 5) importantly, age may be a factor in the response to LRAs. Our asthmatic subjects who received zafirlukast were generally older (median age 42 years), than in most other studies of LRAs, which was just fortuitous.

A recent retrospective analysis in subjects over the age of 50 years demonstrated actual worsening of lung function and an increased exacerbation rate on zafirlukast therapy. [18] The same appeared true, but to a lesser extent, in subjects over the age of 40. Masking of airway inflammation was one of the explanations put forward, but our study found no evidence for this. A further suggestion of potential tolerance comes from a Cochrane systematic review of ICS *versus *LRA that demonstrated a substantially increased risk of exacerbations with LRAs over treatment periods longer than 12 weeks, although increased exacerbations were already apparent even after only 4–8 weeks LRA therapy. [33] This risk seemed highest with zafirlukast compared to montelukast. Unfortunately, the reviewers did not explore these observations and failed to consider tolerance or a potential age effect.

Our prospective data are very suggestive of a rebound deterioration on cessation of drug – this would be highly supportive of true tachyphylaxis and the increase in circulating neutrophils is concerning. Our reading of the literature would suggest that these potential problems with LRAs have not been looked for in a comprehensive fashion despite several large studies and their current widespread use. The possibility that age may influence the effects of reverse agonist activity and dynamic receptor expression is of particular concern and warrants further specific assessment.

## Competing interests

None of the authors have a conflict of interest. The corresponding author Dr David Reid had access to all the data in the study and had final responsibility for the decision to publish.
